# Reconstruction of large-size abdominal wall defect using biodegradable poly-p-dioxanone mesh: an experimental canine study

**DOI:** 10.1186/1477-7819-12-57

**Published:** 2014-03-14

**Authors:** Kenan Huang, Xinyu Ding, Benbo Lv, Linyun Wei, Juxian Sun, Zhifei Xu, Xiong Qin, Hua Tang

**Affiliations:** 1 Department of Thoracic and Cardiovascular Surgery, Changzheng Hospital, the Second Military Medical University, 415 Fengyang Road, Shanghai 200003, China; 2 Department of Thoracic and Cardiovascular Surgery, First People's Hospital of Shanghai, Shanghai Jiaotong University, Shanghai China

**Keywords:** Abdominal wall defect, Canine model, Large size, Marlex, Mesh, Poly-p-dioxanone, Reconstruction

## Abstract

**Background:**

Reconstruction of large-size abdominal wall defect (AWDs) is a huge challenge faced in current surgical practice. In this study, we aimed to evaluate the effectiveness and safety of biodegradable poly-p-dioxanone (PDO) mesh for reconstructing large-size AWDs in an experimental canine model.

**Methods:**

Eighteen experimental canines were randomly and equally divided into three groups, namely, a PDO group, a Marlex group and a control group (*n* = 6 each). Following the creation of a 6 cm × 5.5 cm AWD, PDO mesh and Marlex mesh were used to reconstruct the defect in the PDO and Marlex groups, respectively. The defect was closed using relaxation sutures alone in the control group. Animals were killed 24 weeks after surgery, and reconstruction outcomes were evaluated using radiography, histology and biomechanical testing.

**Results:**

All animals except those in the control group survived the experiment. The PDO group showed no wound dehiscence, herniation or infection, whereas the animals in the Marlex group exhibited marked foreign body reactions. The PDO group had less intraabdominal adhesion than the Marlex group. As shown by radiography, histology and biomechanical testing, PDO mesh exhibited complete degradation and favorable biochemical strength at 24 weeks postsurgery.

**Conclusions:**

PDO mesh implantation is an effective, safe treatment modality for reconstructing large-size AWDs.

## Background

Abdominal wall defects (AWDs) occur mainly after abdominal wall trauma or tumor resection and occasionally as congenital malformations. Small-size AWDs can be closed easily using the residual abdominal wall soft tissues; however, reconstruction of large-size AWDs normally require the use of prostheses and remain a huge challenge in current general surgical practice. The challenges are the hernias with rings greater than 10 cm in diameter, which are more likely to recur [1-4].

It is essential to optimize the choice of mesh materials for successful AWD reconstruction. A series of synthetic, nondegradable mesh prostheses, such as Marlex [5] and polypropylene, have been examined in preclinical and clinical studies. However, use of polypropylene for reconstructing large-size AWDs can lead to some complications, such as chronic pain, abdominal wall stiffness, mesh dislocation and wound fistulas [6,7]. This material is also reported to be associated with a high risk of intraabdominal adhesion, which may lead to ileal and enteric fistulas [8-10]. Developments in materials science and technology have led to the evolution of mesh prostheses into biodegradable material, such as human acellular dermal matrix [11] and small-intestine submucosa [12]. These two materials are derived from human or animal tissue and are beneficial for tissue regeneration [11,12], but they are primarily disadvantageous with regard to fast reabsorption and poor long-term mechanical strength. Furthermore, the cost of these biological meshes is 10 to 70 times greater than the cost of synthetic meshes, and some researchers have reported mixed results with regard to efficacy [13-16]. Composite meshes with protease-treated bovine skin collagen (atelocollagen) are currently available, but increased infection susceptibility and long-term coating failure have been reported [17,18]. Rapid degradation of biodegradable materials compromises the mechanical stability and consequently limits the use of these materials in large-size AWD reconstruction.

Poly-p-dioxanone (PDO) is a colorless, crystalline, resorbable polymer that is degraded by hydrolysis and completely metabolized in the body. It is available in different thicknesses (0.15 mm perforated, 0.25 mm unperforated and 0.55 mm unperforated), is flexible but can preserve its shape, and can be fixed to cartilage with sutures. Thinner plates are resorbed within 25 weeks, and thicker plates are resorbed within 8 months [19,20]. The PDO mesh has been used for many years in chest wall reconstruction and orbital floor reconstruction. Researchers have demonstrated that it offers many advantages, such as excellent flexibility and elasticity, as well as suitable biocompatibility, which induces a minimal inflammatory response [21-24].

In our previous study, we attempted to use PDO mesh for chest wall defect reconstruction [25]. Our results showed that PDO mesh was superior to conventional bioabsorbable polymers mainly in terms of flexibility and elasticity. Moreover, this material also exhibited an appropriate reabsorption rate matching that of soft-tissue regeneration. A knowledge gap exists regarding the use of PDO mesh in large-size AWD reconstruction; therefore, we conducted an experimental canine study to evaluate the effectiveness and safety of PDO mesh alone in reconstructing large-size AWDs.

## Methods

### Preparation of poly-p-dioxanone mesh

**Figure 1:**
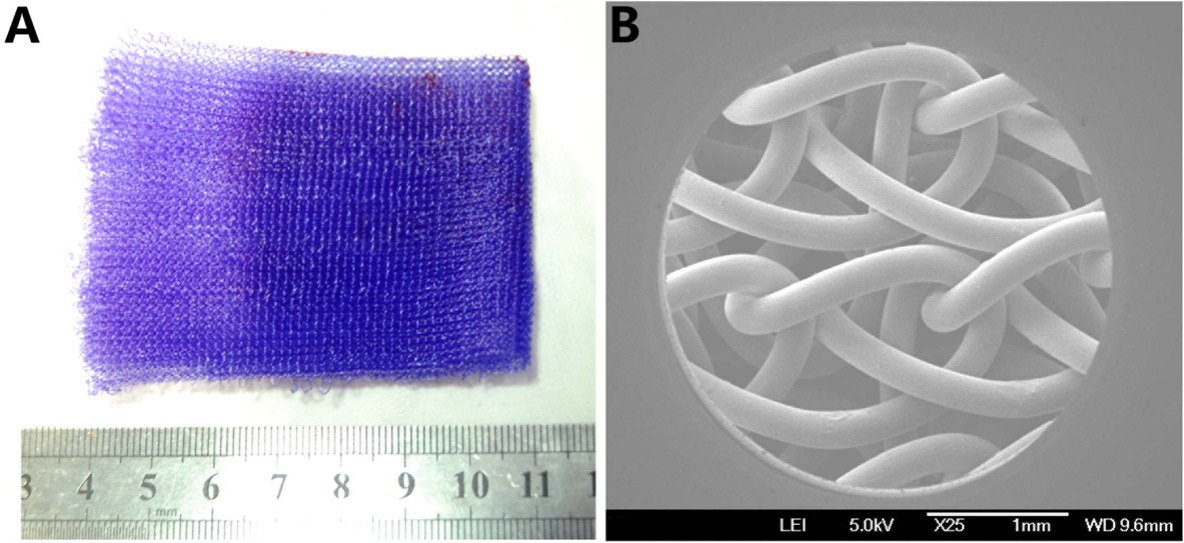
**Poly-p-dioxanone mesh with a pore size of 0.2 mm. (A)** Gross appearance. **(B)** Scanning electron microscopy image (original magnification, ×25).

Biodegradable PDO threads (Samyang, Seoul, South Korea) at a diameter of 0.8 mm were weaved into a mesh 7 cm in length and 6.5 cm in width at Donghua University, Shanghai, China. A scanning electron microscope was used for mesh ultrastructural analysis (Figure 1).

#### Laboratory animals

The study protocol was approved by the Animal Care and Use Committee of the Second Military Medical University, Shanghai, and carried out in accordance with the latest version of the US National Institutes of Health’s Guide for the Care and Use of Laboratory Animals. Eighteen adult mongrel dogs of either sex, ages 1 to 2 years and weighing 15 to 18 kg were bred and housed at the Center for Laboratory Animals at the Second Military Medical University. Animals were randomly and equally divided into three groups according to the type of repair to be performed: a PDO group, a Marlex group (crystalline polypropylene and high-density polyethylene; Jia Te Plastics Co, Dongguan, China) and a control group (*n* = 6 for each group). The first two groups underwent large-size AWD reconstruction using PDO mesh or Marlex mesh, respectively and the control group underwent defect closure surgery without the use of any mesh prosthesis.

#### Surgical procedure

**Figure 2:**
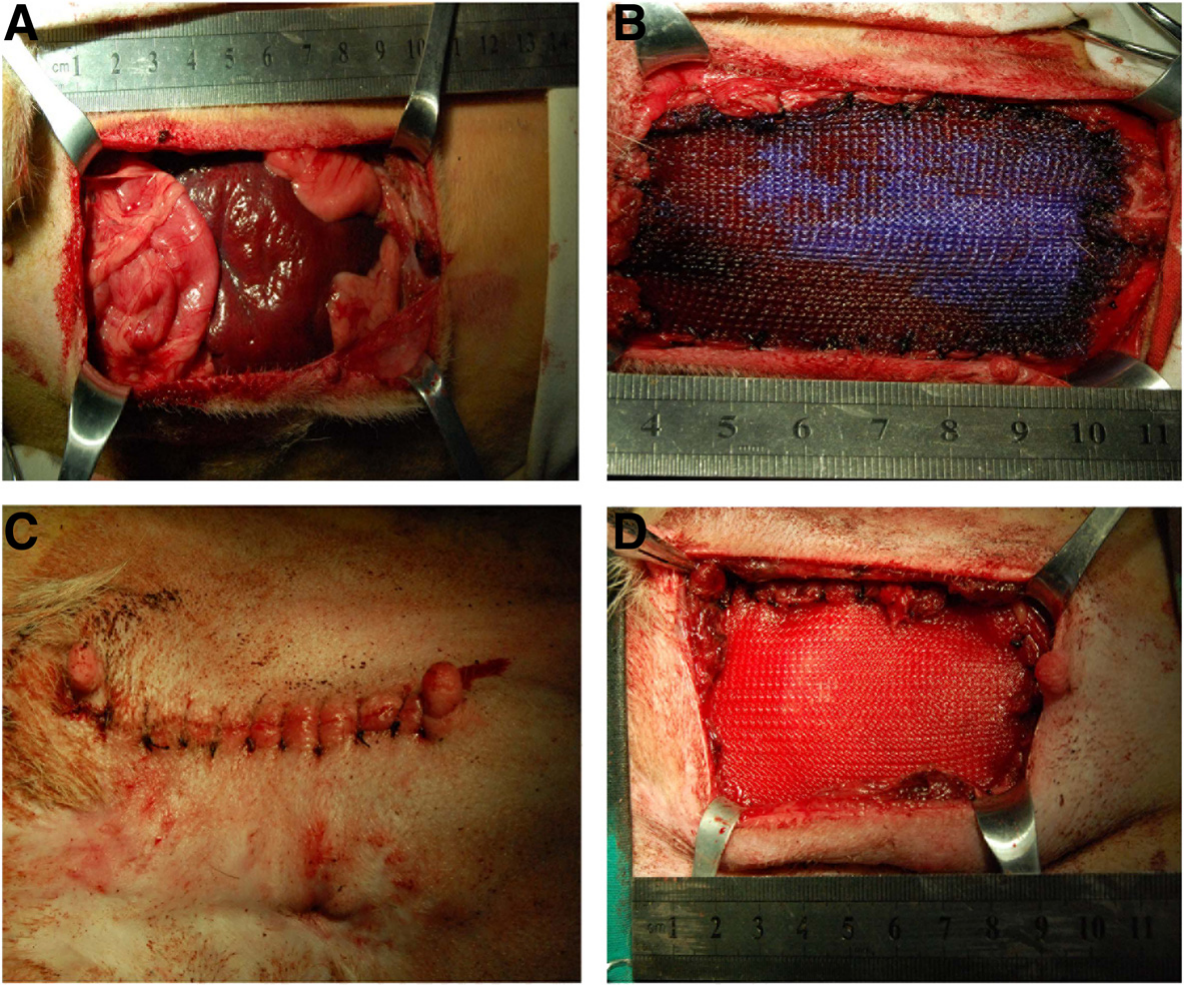
**Surgical procedure performed for large-size abdominal wall defect reconstruction.** A 6 cm × 5.5 cm abdominal wall defect (AWD) **(A)** was created by removing bilateral rectus abdominis muscles. The AWD was reconstructed using poly-p-dioxanone mesh **(B)** or Marlex mesh **(C)**. **(D)** The defect was closed using relaxation sutures in the control group.

An intravenous injection of pentobarbital sodium (30 mg/kg; Shanghai Suolaibao Biotechnology, Shanghai, China) was given to induce general anesthesia, and an intravenous infusion of ketamine hydrochloride (2 mg/kg; Shanghai Suolaibao Biotechnology) and atracurium besylate (0.3 mg/kg; Shanghai Suolaibao Biotechnology) was given to maintain general anesthesia throughout the procedure. The animals were subsequently placed in the supine position while under general anesthesia with endotracheal intubation. The abdominal wall skin was shaved, sterilized with povidone-iodine (Shanghai Suolaibao Biotechnology) and draped as routinely done. A full-thickness midline xyphopubic AWD (6 cm × 5.5 cm) was created, by which the fascia, underlying rectus abdominis muscle and peritoneum were resected (Figure 2A). In the PDO or Marlex mesh group, the mesh (7 cm × 6.5 cm) was placed intraabdominally with a 0.5-cm overlap and fixed tension-free to the abdominal wall with 2-0 polypropylene sutures (Ningbo Medical Needle Co, Ltd, Ningbo, China). Subsequently, the abdominal wall fascia was closed at the midline with a running 2-0 polypropylene suture, and the subcutis and skin were closed with interrupted 2-0 polypropylene sutures (Figures 2B and 2C). The defect was closed using relaxation sutures alone in the control group (Figure 2D). The endotracheal tube was removed when the animal resumed spontaneous breathing. After surgery, the animals were given an analgesic (1 ml of buprenorphine) and an intramuscular injection of prophylactic 1,600,000-U procaine benzylpenicillin (Shanghai Suolaibao Biotechnology) and 80,000-U gentamicin sulfate (Shanghai Suolaibao Biotechnology) for the first three successive days. Animals were housed in single cages with a 12-hour day–night cycle, fed a commercially available diet and given free access to water. The animals were killed with an intravenous injection of 7,000 mg of pentobarbital sodium (Shanghai Suolaibao Biotechnology) and evaluated for clinical recurrence.

#### Radiographic examination

To further analyze the process of mesh degradation, abdominal computed tomography (CT) scans were taken at 12 and 24 weeks following AWD reconstruction while the animals were maintained under general anesthesia. Image processing and three-dimensional reconstruction were accomplished using the Advantage Workstation 4.2 (GE Healthcare, Shanghai, China).

### Gross evaluation of wound adhesion

The animals were killed at 24 weeks following AWD reconstruction. Gross wound adhesion was evaluated using a validated semiquantitative visual analogue scale ranging from a minimum of 0 to a maximum of 3 points [26,27], with 0 signifying no significant adhesion; 1 indicating a thin, narrow, easily detachable adhesion; 2 meaning a thick adhesion limited to a single area; and 3 meaning a thick, broad adhesion involving the anterior or posterior abdominal wall and the viscera.

#### Biomechanical test

Freshly harvested regenerated soft-tissue samples were loaded onto a tensile testing machine (School of Materials Science and Engineering Lab, Jiao Tong University, Shanghai, China) for tensile strength measurement with the speed calibrated at 100 mm/min at 20°C. The load rate was set at 0.5 N/mm, and the primary load was 1.5 N. A stress–strain curve was plotted and fitted to produce the tensile strength and elastic modulus.

#### Histological examination

Regenerated soft-tissue samples were fixed in 10% formaldehyde (Mengzhuang Bio-Technology Co, Ltd, Beijing, China) for 72 hours and decalcified in 15% formic acid (Mengzhuang Bio-Technology Co, Ltd) for 2 to 6 weeks. Tissue samples were embedded in paraffin and cut into 5-μm-thick sections with a microtome for hematoxylin and eosin staining according to routine procedure. Native abdominal wall soft tissue was examined using the same protocol used for the controls.

#### Statistical analysis

The SPSS version 16.0 software (IBM SPSS, Chicago, IL, USA) was used for statistical analysis. All data are expressed as mean ±SD. The means were compared using one-way analysis of variance, and the two independent samples were analyzed by Student’s *t*-test. *P* < 0.05 was considered statistically significant.

## Results

**Figure 3:**
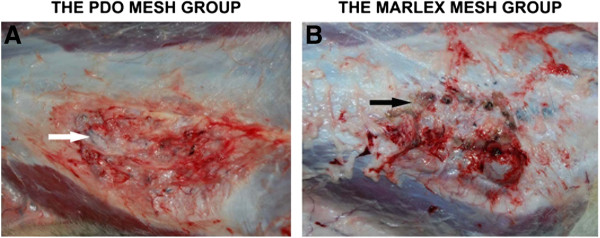
**Macroscopic appearance of reconstructed abdominal wall defects at 12 weeks. (A)** The animals in the poly-p-dioxanone group exhibited good wound-healing (white arrow). **(B)** One animal in the Marlex group had a remarkable foreign body reaction and wound dehiscence (black arrow).

In the control group, all animals died immediately after surgery as a result of herniation. The animals in the other two groups survived the surgeries. The surviving animals exhibited good general well-being and normal activities at 4, 12 and 24 weeks postsurgery. All the surviving animals exhibited no wound dehiscence, herniation or infection (Figure 3A), except for one dog in the Marlex group that had a marked wound foreign body reaction and dehiscence (Figure 3B).

**Figure 4:**
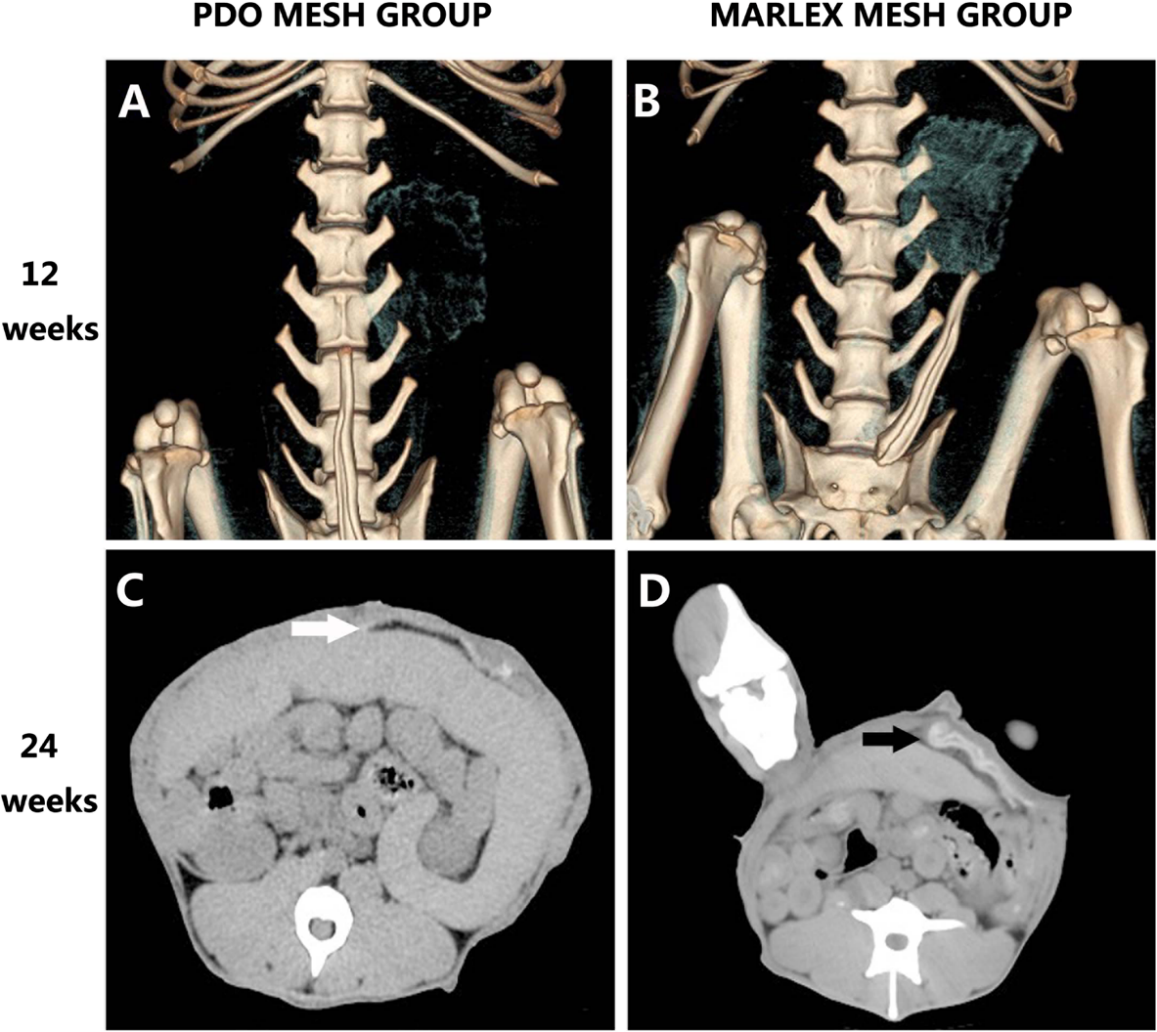
**Three-dimensional reconstructions of abdominal computed tomography scans.** At 12 weeks, The poly-p-dioxanone (PDO) mesh **(A)** and the Marlex mesh **(B)**. At 24 weeks, On abdominal CT scans, the poly-p-dioxanone (PDO) mesh (black arrow in (**C**)) and the Marlex mesh (white arrow in (**D**)).

On abdominal CT scans, the radiopacity of the PDO mesh decreased but could still be observed at 12 weeks (Figure 4A). In contrast, Marlex mesh remained radiopaque throughout the 12 weeks after surgery (Figure 4B). This radiopacity had mostly disappeared at 24 weeks, indicating that the PDO mesh had nearly completely degraded (Figure 4C). Furthermore, we observed obvious shrinkage in the Marlex mesh group, but not in the PDO mesh group (Figure 4D).

**Figure 5:**
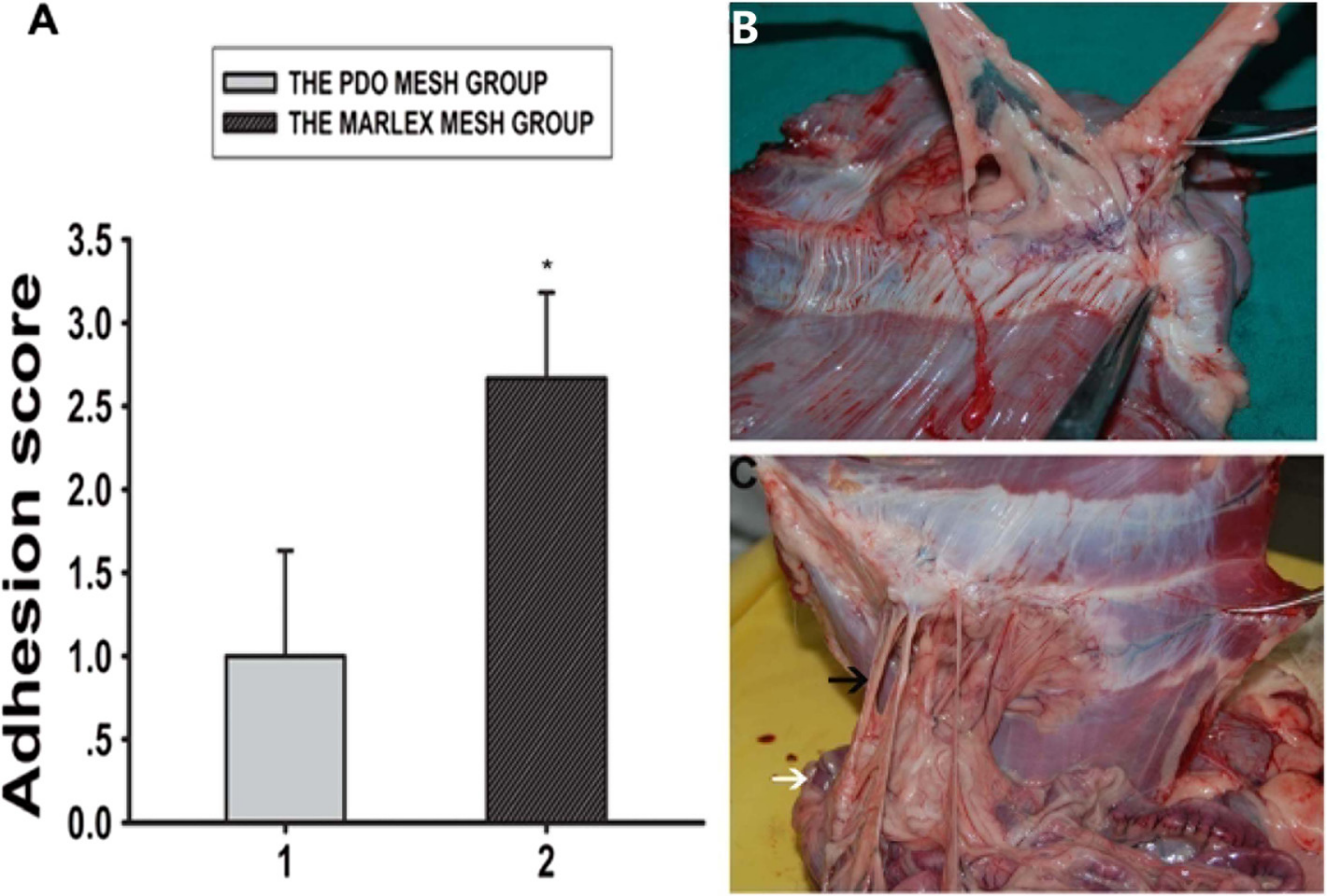
**Macroscopic evaluation of wound adhesion of reconstructed abdominal wall at 24 weeks. (A)** Overall, poly-p-dioxanone (PDO) mesh had a significantly lower gross wound adhesion score than Marlex mesh (*P < 0.05). **(B)** All PDO meshes became completely degraded at 24 weeks postsurgery with easily detachable adhesions to the peritoneum and the omentum. **(C)** Marlex mesh showed extensive dense adhesions to the omentum and the visceral organs, including the omentum and the colon. The black and white arrows indicate the omentum and the colon, respectively.

The proportion of adhesion was recorded for each group. Statistical analysis of the adhesion scores was then performed. The mean adhesion score of the PDO mesh group was 1.1 ±0.2, and the mean Marlex mesh group score was 2.6 ±0.2. Overall, PDO mesh had a significantly lower gross wound adhesion score than Marlex mesh (*P* < 0.05) (Figure 5A). All PDO meshes became completely degraded at 24 weeks postsurgery with easily detachable adhesions to the peritoneum and the omentum (Figure 5B). Marlex mesh showed no marked degradation, but had extensive dense adhesion to the omentum and the visceral organs, including the omentum and the colon (Figure 5C).

**Figure 6:**
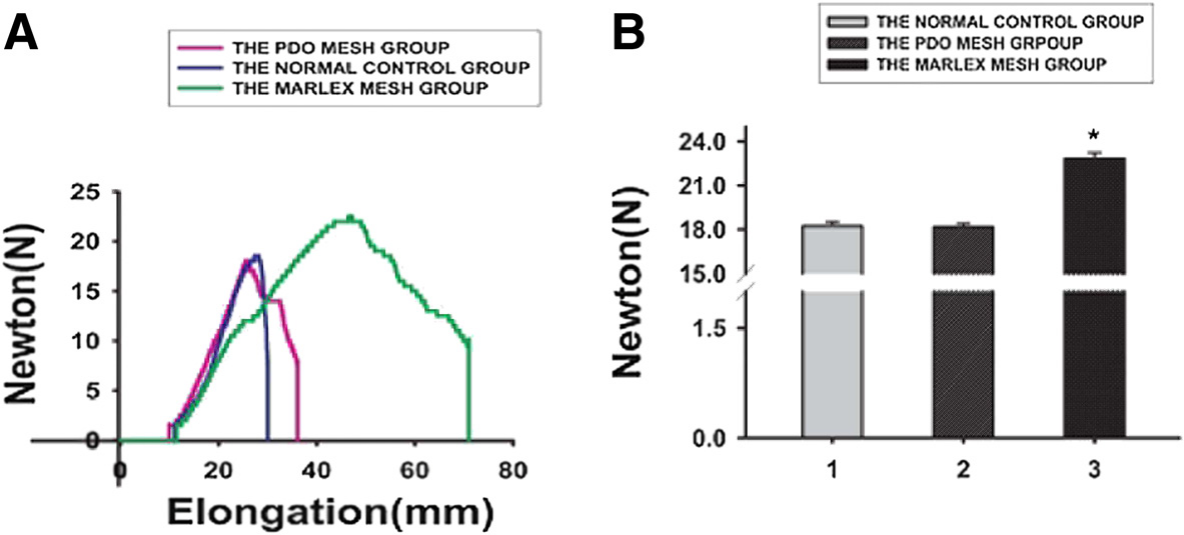
**Mechanical properties of implanted biomaterials at 24 weeks after surgery. (A)** Ultimate tensile strength of each group. **(B)** The statistical analysis of the ultimate tensile strength. Data are mean ±SD (*n* = 6). *P* < 0.05. PDO, Poly-p-dioxanone.

To compare the normal abdominal wall and evaluate the mechanical properties of the reconstructed abdominal wall, the tensile strength of the implanted biomaterials was measured at 24 weeks after surgery. The Marlex group had significantly greater tensile strength than the PDO group and the control group (PDO vs. Marlex vs. control, 22.8 ±0.4 N vs. 18.2 ±0.3 N vs. 18.3 ±0.3 N; *P* = 0.000), whereas the PDO group exhibited tensile strength similar to that of the control group (*P* = 0.664) (Figures 6A and 6B).

**Figure 7:**
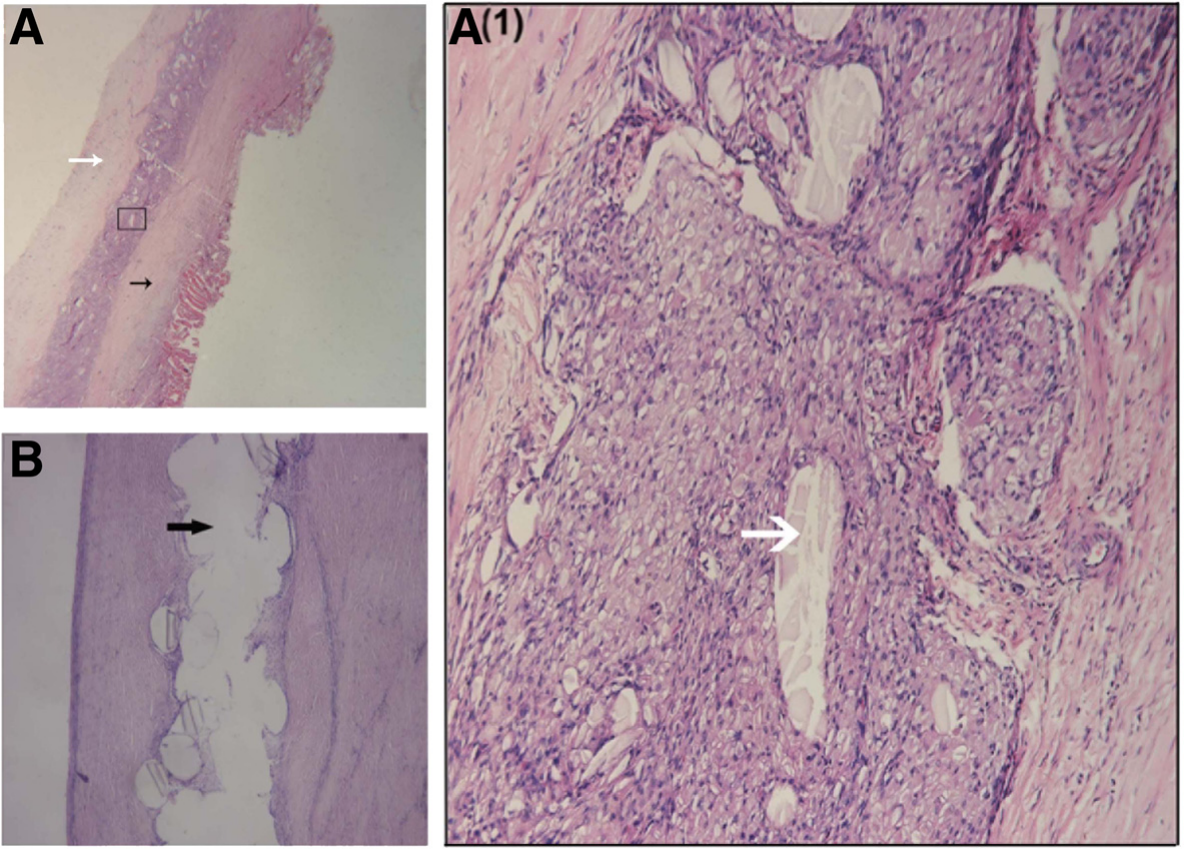
**Histological images of the poly-p-dioxanone and Marlex meshes. (A)** The white and black arrows indicate the inner and outer sides of the reconstructed abdominal wall, respectively. **(A(1))** Detailed, high-magnification image of the boxed area in **(A)** (original magnification, ×100). The white arrow points to the residual poly-p-dioxanone (PDO) mesh. **(B)** The black arrow indicates the absence of soft-tissue regeneration in the Marlex mesh. Images in **(A)** and **(B)** were stained with hematoxylin and eosin (original magnification, ×10).

PDO mesh exhibited almost complete degradation on histological examination at 24 weeks postsurgery, and a small amount of mesh residuals were enveloped by the regenerated soft tissues and surrounded by extensive fibroconnective tissues (Figure 7A). Marlex mesh showed no microscopic degradation either (Figure 7B).

## Discussion

An optimal mesh prosthesis for reconstructing AWDs should be easily fashioned but resistant to moisture, disinfection or mechanical tension; should not be immunogenic or carcinogenic; and should not cause chemical, inflammatory, foreign body, allergic or hypersensitivity reactions [26]. None of the currently available prosthesis materials meet all the aforementioned requirements, although nondegradable materials remain the mainstay choice for large-size AWD reconstruction. However, use of these nonbiological, nondegradable prostheses, such as alloy fabrics and high-molecular-mass polymers, carries a high risk of long-term complications.

Biological materials derived from autologous tissues (fascia, muscle flap and autogenous dermis) [28], allogeneic tissue (amniotic membrane) [29] and xenogeneic tissues (porcine heart valve and bovine peritoneum) [30] are beneficial for tissue regeneration in the recipient site with a relatively lower long-term risk than nonbiological materials. However, AWD reconstruction using allograft is associated with a high risk of herniation recurrence, mainly due to reduction in biological mesh tensile strength and increase in elastin content in human dermis [29]. Moreover, biomechanical properties of biological meshes are highly variable between donors [31].

Artificial synthetic mesh was first introduced by Usher in 1958 [5], and it is widely used in general surgical practice because of its favorable biocompatibility and mechanical stability. As a nondegradable material, prosthetic mesh, such as Marlex mesh, is associated with a series of postoperative adverse effects, including persistent pain, hematoma, wound erosion, infection and herniation, and even enterocutaneous fistula [1,32,33]. McKenna *et al*. [34] reported an approximately 25% infection rate associated with Marlex mesh implantation. Serious cases normally require a second-look surgery to remove the infectious prosthesis [34]. A large number of newly emerging tissue engineering materials have been developed for reconstructing human body wall defects in preclinical and clinical studies based on breakthroughs in materials science and technology [35-38]. PDO is a synthetic absorbable polymer that has been used for surgical suture weaving. In our previous experimental canine model study, we demonstrated that chest wall defects can be structurally and functionally reconstructed by using a composite implant containing PDO mesh [37]. In the present study, we also successfully reconstructed large-size AWDs by using PDO mesh alone. To the best of our knowledge, this study is the first to report the use of synthetic absorbable polymer alone for reconstructing experimental large-size AWDs.

In our study, PDO mesh showed good biocompatibility. No animals in the PDO group had wound dehiscence, herniation or infection, whereas one animal exhibited remarkable foreign body reaction and consequent wound nonhealing in the Marlex group. Furthermore, histological evidence showed that PDO mesh expedited soft-tissue regeneration by inducing collagen deposition and neoangiogenesis with a reasonable degradation rate over the course of 24 weeks. In contrast, Marlex mesh showed no signs of absorption or degradation within this period. Researchers in similar previous studies [13] have found that microscopically intact polypropylene mesh filaments were surrounded by variably organized fibrous tissue, and, as expected, a pronounced foreign body reaction and intraabdominal adhesion formation.

Tissue adhesion usually occurs following fibrin exudation in the wound. Our results show that PDO mesh implantation caused only mild wound adhesion compared to Marlex mesh. As a degradable material, PDO mesh is expected to elicit minimal foreign body reaction and inflammatory response. Shrinkage in mesh implants is a major adverse effect, regardless of the use of degradable or nondegradable material. This reduction results mainly from chronic scarring and scar retraction and consequent distortion or dislocation of the implanted mesh. The presence of an irregularly shaped mesh surface will induce foreign body and inflammatory reactions, which are destructive of the newly regenerated tissue and makes tissue prone to infection and the formation of fistula. Our follow-up CT scans showed that remarkable mesh shrinkage occurred in the Marlex group, but not in the PDO group. Previous histological studies have also confirmed that PDO mesh implantation was associated with less serious inflammation and fibrosis, formation of smaller granulomas and less cell turnover and remodeling [39]. More importantly, once the initial infection has developed, only antibiotic gauze needs to be applied to the wound during dressing change. For synthetic, nondegradable mesh prostheses, however, the mesh has to be taken off. When bacteria enter the surgical incision, the development of a mesh infection is dependent on the bacteria adherent to the prosthetic material. Only a clean wound can heal rapidly and without infection.

A major technical concern regarding biodegradable mesh prostheses is long-term biomechanical strength. No wound herniation occurred in the PDO group over the course of 24 weeks in our present study. Postoperative 4-week follow-up CT scans showed that PDO mesh, just as nondegradable Marlex mesh, maintained a well-preserved shape and provided adequate mechanical support for AWD reconstruction at an early phase. Furthermore, 12- and 24-week postoperative CT scans suggested that PDO mesh-regenerated tissue had sufficient tensile and burst strength to withstand abdominal wall tension, although the mesh had become degraded. Biomechanical testing results also confirmed that PDO mesh–regenerated tissue had favorable mechanical strength similar to the native abdominal wall in the midterm sense, although it was even higher in Marlex mesh.

This study has some limitations. First, for the protection of the animals, this study had a relatively small sample size, with only six animals in each group. Second, the follow-up period was only 24 weeks, and therefore it remains unknown whether PDO mesh implantation was effective and safe for large-size AWD reconstruction. In addition, the PDO mesh was not found to be biodegraded completely at 24 weeks postsurgery, hence it is still likely to have retained sufficient tensile strength to prevent failure at the surgical site and subsequent herniation. Therefore, long-term observation is necessary in future research. Third, PDO mesh could still induce some intraabdominal adhesions. The most probable cause is the fact that our PDO mesh was not coated with any natural macromolecular biomaterial. It is known that additional coating with artificial or biological polymers, such as collagen, can effectively prevent the formation of postoperative wound adhesions [40-42].

## Conclusions

PDO mesh is a good alternative to Marlex mesh for large-size AWD reconstruction. PDO mesh has a reasonable degradation rate, good biocompatibility and sufficient biomechanical strength similar to the native abdominal wall in rectus abdominis. The effectiveness and safety of using PDO mesh in reconstructing large-size AWD needs to be validated in further long-term preclinical and clinical studies.

## Competing interests

The authors declare that they have no competing interests.

## Authors’ contributions

HT participated in the design of the study. BLv and LW performed the statistical analysis. JS interpreted the data. XD participated in its design and coordination and helped to draft the manuscript. KH participated in the sequence alignment and drafted the manuscript. XQ critically revised the manuscript for important intellectual content. All authors read and approved the final manuscript.
